# Mechanisms to increase propulsive force for individuals poststroke

**DOI:** 10.1186/s12984-015-0030-8

**Published:** 2015-04-18

**Authors:** HaoYuan Hsiao, Brian A Knarr, Jill S Higginson, Stuart A Binder-Macleod

**Affiliations:** Biomechanics and Movement Science Program, University of Delaware, 540 S. College Avenue, Suite 201F, Newark, DE 19716 USA; Delaware Rehabilitation Institute, Newark, DE 19716 USA; Department of Mechanical Engineering, University of Delaware, Newark, DE 19716 USA; Department of Physical Therapy, University of Delaware, Newark, DE 19716 USA

**Keywords:** Stroke, Gait, Propulsion, Speed, Ankle moment, Trailing limb angle

## Abstract

**Background:**

Propulsive force generation is critical to walking speed. Trialing limb angle and ankle moment are major contributors to increases in propulsive force during gait. For able-bodied individuals, trailing limb angle contributes twice as much as ankle moment to increases in propulsive force during speed modulation. The aim of this study was to quantify the relative contribution of ankle moment and trailing limb angle to increases in propulsive force for individuals poststroke.

**Methods:**

A biomechanical-based model previously developed for able-bodied individuals was evaluated and enhanced for individuals poststroke. Gait analysis was performed as subjects (N = 24) with chronic poststroke hemiparesis walked at their self-selected and fast walking speeds on a treadmill.

**Results:**

Both trailing limb angle and ankle moment increased during speed modulation. In the paretic limb, the contribution from trailing limb angle versus ankle moment to increases in propulsive force is 74% and 17%. In the non-paretic limb, the contribution from trailing limb angle versus ankle moment to increases in propulsive force is 67% and 22%.

**Conclusions:**

Individuals poststroke increase propulsive force mainly by changing trailing limb angle in both the paretic and non-paretic limbs. This strategy may contribute to the inefficiency in poststroke walking patterns. Future work is needed to examine whether these characteristics can be modified via intervention.

## Background

Current gait rehabilitation for individuals poststroke focuses on increasing gait velocity because it is a powerful indicator of function and prognosis after stroke [1]. Walking speed has been shown to be associated with community walking ability, and an increase in gait velocity that produces a transition to a higher level of ambulation results in better community participation and quality of life [1]. Because walking speed is also a reliable and responsive measurement, many recent clinical trials that target improved walking use walking speed as a primary outcome measure [2]. Thus, aiming to maximize walking speed is commonly a therapeutic goal.

Previous studies have shown that walking speed is related to propulsive force, defined as the anterior component of the ground reaction force (AGRF) during gait [3]. More importantly, a recent study showed that improvements in paretic propulsive force are correlated to changes in self-selected walking speed and changes in fastest comfortable walking speed following a 12-week locomotor intervention [4]. Thus, paretic propulsive force can be modified through intervention and is related to the improvement in walking speed. Understanding the mechanism to increase propulsive force would allow for the design of rehabilitation strategies for improving paretic propulsion and ultimately lead to increase walking speed.

There are two critical factors for propulsive force generation: ankle moment and the position of the center of pressure (COP) relative to the body center of mass (COM) [5]. Peterson et al. showed that ankle moment is correlated to propulsive force for able-bodied individuals and in the non-paretic leg for individuals poststroke [5]. This finding is consistent with previous studies that showed ankle plantarflexor muscle activity is associated with the propulsive force in the paretic limb [6] and that ankle moment is related to walking speed [7]. Another critical predictor for propulsive force is the position of the COP relative to the body COM. This relative position affects the orientation of the ground reaction force (GRF) vector and, therefore, determines the proportion of the GRF being distributed anteriorly. Tyrell et al measured trailing limb angle (TLA), defined as the angle between the lab’s vertical axis and the vector from the 5^th^ metatarsal joint to the great trochanter, and found that stroke survivors increased peak TLA and propulsion as walking speed progressively increased [8]. Similarly, another study measured the angle between the vertical and the vector from the COM of the foot to the COM of the pelvis. They found that this angle is an important predictor and is positively related to propulsive force during able-bodied and hemiparetic walking [5]. Using a simplistic quasi-static model, our lab has determined the relative contribution of ankle moment and TLA to propulsive force in able-bodied individuals [9]. We showed that the TLA contributes almost twice as much as ankle moment to increases in propulsive force when able-bodied individuals increase their walking speeds [9]. However, individuals poststroke may adopt different strategies to increase their propulsive force compared with able-bodied individuals. The purpose of this study was to test the accuracy of our previous model and to quantify the relative contribution of ankle moment and TLA to increases in propulsive force for individuals poststroke.

## Methods

A total of 24 individuals poststroke participated in this study (10 female; 15 left hemiparetic; average age 60 years; body weight 89 kg; stroke onset 5 years). Exclusion criteria included congestive heart failure, peripheral artery disease with claudication, diabetes not under control via medication or diet, shortness of breath without exertion, unstable angina, resting heart rate outside the range of 40 to 100 beats per minute, resting blood pressure outside the range of 90/60 to 170/90 mm Hg, inability to communicate with the investigators, pain in lower limbs or spine, total knee replacement, cerebellar involvement, and neglect (star cancellation test). Subjects that walked with a negative TLA at the instant of peak AGRF were also excluded from this study. This study was approved by the Institutional Review Board of the University of Delaware and each subject provided written informed consent for participation in this study.

### Experimental procedure

Each subject walked at their self-selected (SS) and fast (FS) walking speeds wearing a safety harness that provided no body weight support. For safety, subjects were allowed to use a handrail located at the side of the treadmill. Verbal instructions on using the handrail as minimal as possible were provided. SS walking speed was defined as the subject’s comfortable walking speed and FS walking speed was the fastest speed that subjects could maintain for 4 minutes of continuous walking. Gait analysis was performed on an instrumented split-belt treadmill (Bertec Corp., Columbus, OH, USA) recording three dimensional forces with two embedded 6 degree-of-freedom force plates capturing at 1080 Hz. Kinematic data were recorded with a 62 marker set and eight camera passive motion capture system that detects motion of the reflective markers at 60 Hz (Motion Analysis Corp., Santa Rosa, CA, USA). Data processing was completed using Cortex and Visual 3D (C-Motion Inc., Bethesda, MD, USA). Kinematic data were filtered using a bi-directional Butterworth low-pass filter at 6 Hz.

Peak AGRF (*F*_*a*_) was defined as the maximum AGRF during stance between the onset of the propulsion (anterior) phase of anterior-posterior ground reaction forces and toe-off. Ankle moment (*M*_*a*_) was defined as the ankle plantarflexion moment during stance. TLA was defined as the angle between the laboratory’s vertical axis and the vector joining the greater trochanter with the fifth metatarsal head (see [9] for more detailed description). Handrail forces in the vertical and horizontal directions were analyzed at the instant of peak paretic AGRF during each subject’s fast walking speeds. The AGRF, ankle moment, and TLA at the instant of peak AGRF were used in our model. All data were averaged across strides with 30 seconds trial duration for a given speed.

### Model development and validation

A model previously developed for able-bodied individuals [9] (see *Eq.**1*, where *F*_*a*_ is the AGRF, *M*_*a*_ is the ankle moment, and TLA is the trailing limb angle) was first applied to the data obtained from individuals poststroke in this study. This model was evaluated using data from the paretic and non-paretic leg at SS and FS walking speeds. The model explained between 54-70% of the variance in propulsive force; however, the trendline slopes for the measured versus the predicted propulsive forces were approximately 0.77, indicating that the model over-estimated propulsive force for individuals poststroke.

1 $$ {F}_a=7.013{M}_a sin(TLA) $$

**Figure 1:**
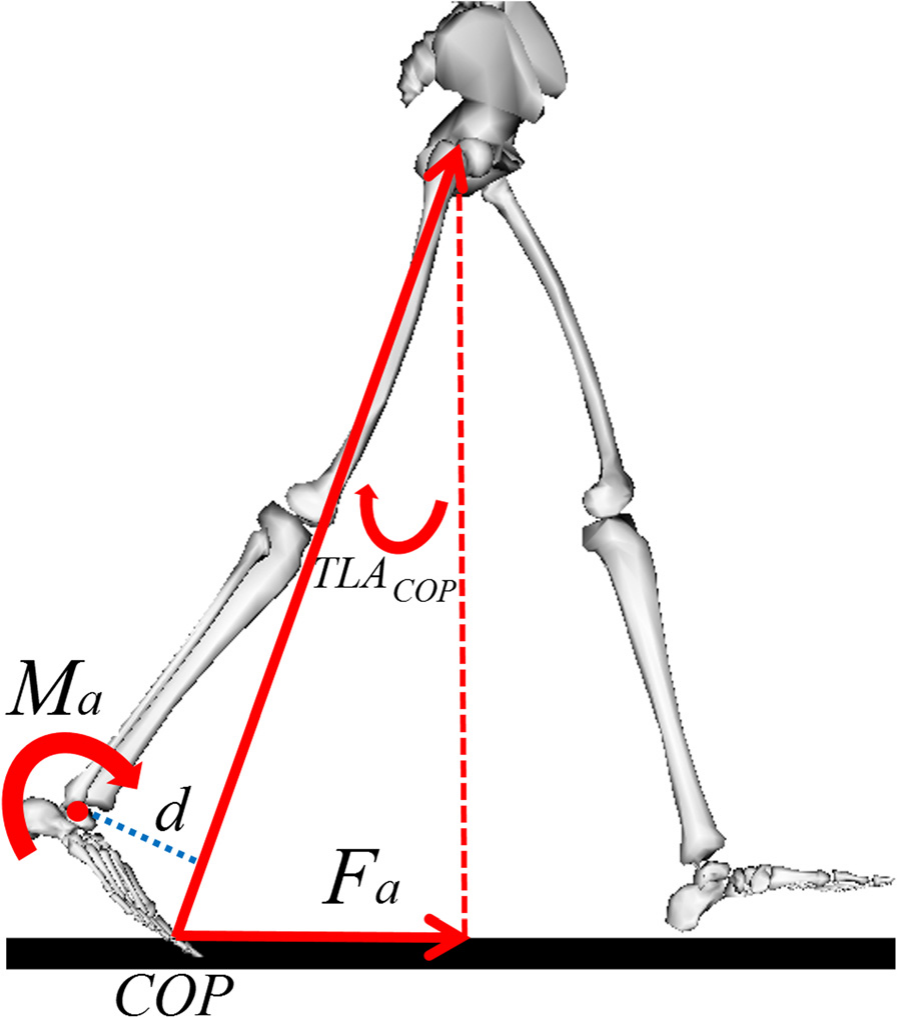
Diagram of variables of interest. ***F***_***a***_ was the anterior component of the ground reaction force. ***M***_***a***_ was the ankle plantarflexion moment. COP was the center of pressure. **TLA**_***cop***_ was measured as the angle between the laboratory’s vertical axis and the vector joining the greater trochanter with the COP. *d* was the perpendicular distance from the ankle joint to the vector joining the greater trochanter with the COP.

Thus, an enhanced model was developed for better accuracy. For the enhanced model, rather than using the constant, 7.013, a variable, *d,* was included to account for the lever arm length of the ground reaction force (Figure 1). In addition, TLA was replaced by TLA*cop*, the angle between the laboratory’s vertical axis and the vector joining the greater trochanter with the COP, to provide a better estimation of the ground reaction force angle (*Eq.**2*).

2 $$ {F}_a=\frac{1}{d}{M}_a sin\left( TL{A}_{cop}\right) $$

Using *Eq.**2*, the increase in propulsive force was calculated:

3 $$ \varDelta {F}_a={F}_{a2}-{F}_{a1}\kern0.5em =\frac{1}{d_2}{M}_{a2} \sin TL{A_{cop}}_2-\frac{1}{d_1}{M}_{a1} \sin TL{A_{cop}}_1, $$

where “∆” denotes the change from the SS to the FS session, subscript “_1_” denotes the value at the SS session and subscript “_2_” denotes the value at the FS session. Letting, sin *TLA*_2_ = sin *TLA*_1_ + Δ sin *TLA*, *M*_*a*2_ = *M*_*a*1_ + *ΔM*_*a*_, *and d*_2_ = *d*_1_ + Δ*d*, we get

4 $$ \Delta {F}_a=\frac{1}{d_1+\Delta d}\left[\left({M}_{a1}+\Delta {M}_a\right)\left( sinTL{A_{cop}}_1+\Delta sinTL{A}_{cop}\right)\right]-\frac{1}{d_1}{M}_{a1} sinTL{A_{cop}}_{{}_1} $$

Rearranging Eq.4, we get

5 $$ \Delta {F}_a=\frac{1}{d_1+\Delta d}\left[{M}_{a1}\Delta \sin TL{A}_{cop}+\Delta {M}_a \sin TL{A_{cop}}_1+\Delta {M}_a\Delta \sin TL{A}_{cop}-\left(\frac{\varDelta d}{d_1}\right){M}_{a1} \sin TL{A_{cop}}_{{}_1}\right] $$

Based on Eq.5, four components contribute to the change in propulsive force: $\frac{1}{d_1+\Delta d}{M}_{a1}\Delta \sin TL{A}_{cop}$, $\frac{1}{d_1+\Delta d}\Delta {M}_{a1} \sin TL{A_{cop}}_{{}_1}$, $\frac{1}{d_1+\Delta d}\Delta {M}_a\Delta \sin TL{A}_{cop}$, and $-\frac{1}{d_1+\Delta d}\left(\frac{\Delta d}{d_1}\right){M}_{a1} \sin TL{A_{cop}}_{{}_1}$. The first component represents the contribution of the changes in TLA to propulsive force. The second component represents the contribution of the changes in ankle moment to propulsive force. The third component represents the contribution from the interaction between changes in TLA and ankle moment. The last component represents the relative contribution from changes in lever arm length to propulsive force. Each of the above terms was calculated and negative values were set to 0 (no contribution). The relative contributions were then calculated by dividing each term by the sum of all terms. Note that $\frac{1}{d_1+\Delta d}$ would have no impact on the relative contributions because it exists in all terms and would be cancelled out during the calculation.

### Model validation and statistical analysis

Pearson’s correlation coefficients (*r*) were calculated by comparing the predicted to the measured peak AGRF to evaluate the ability of the model to predict propulsive force for all subjects at two different speeds. The slopes of the trendlines were calculated by setting the intercepts to 0. In addition, a paired t-test was used to detect whether differences exist between the predicted and the experimental changes in peak AGRF. A 1-tailed paired t-test was used to detect increases in biomechanical measurements from SS to FS. The significance level was set at an alpha of 0.05.

## Results

**Figure 2:**
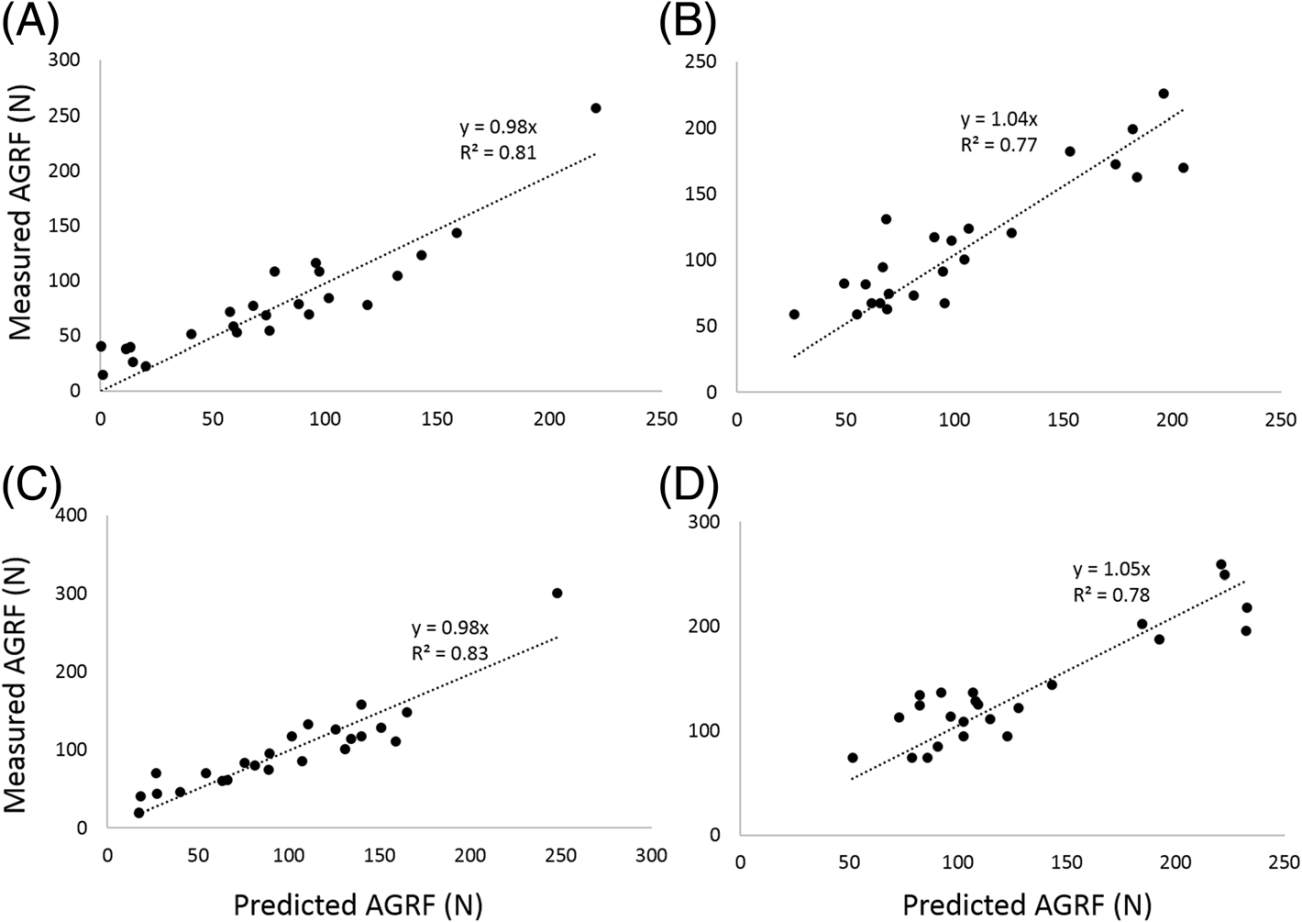
Relationships between the measured and predicted peak anterior ground reaction force (AGRF). **(A)** Paretic propulsion during self-selected walking speed. **(B)** Non-paretic propulsion during self-selected walking speed. **(C)** Paretic propulsion during fast walking speed. **(D)** Non-paretic propulsion during fast walking speed.

**Figure 3:**
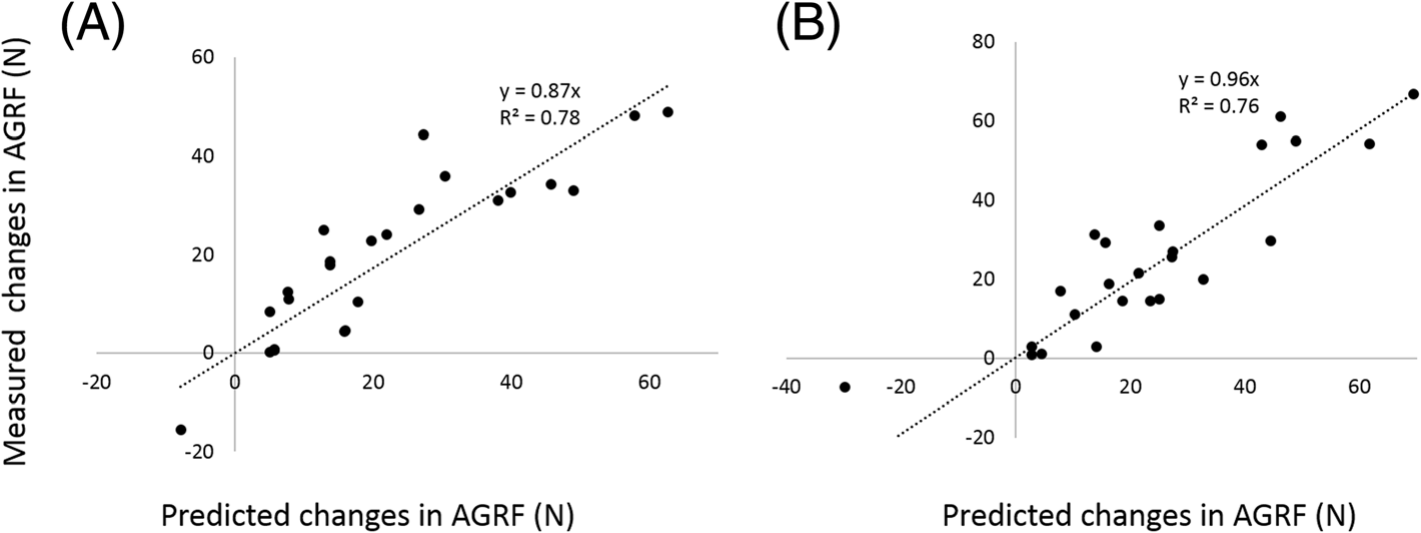
Relationships between the measured and predicted changes in peak anterior ground reaction force (AGRF). **(A)** Changes in paretic propulsion. **(B)** Changes in non-paretic propulsion.

The model predicted peak AGRF was the product of ankle moment and sin (*TLA*_*cop*_) divided by the lever arm length (d) (E*q.**2*). We validated the model in both the paretic and non-paretic leg at SS and FS walking speeds (Figure 2). The enhanced model explained more than 75% of the variance in propulsive force with the trendlines slopes close to 1. Model predicted changes in propulsive force were calculated from Eq.3. This model also explained more than 75% of the variance in changes in propulsive force with speed (Figure 3). No significant differences were found between the predicted (mean: paretic = 22.44 N, non-paretic = 23.88 N) versus the measured (mean: paretic = 20.1 N, non-paretic = 24.97 N) changes in propulsive force (t = 1.34, p = 0.19 for the paretic and t = -0.53, p = 0.6 for the non-paretic).

**Figure 4:**
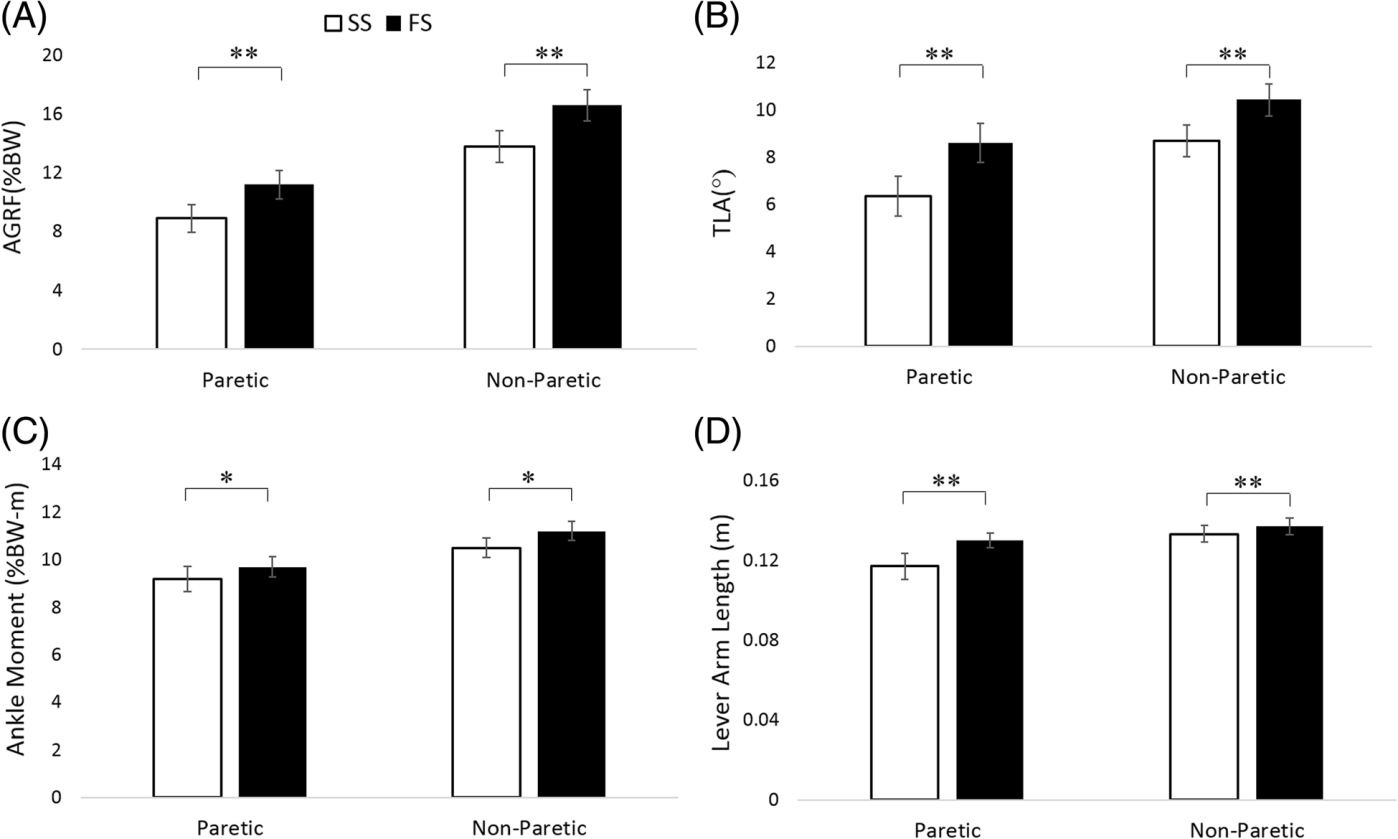
Means and standard errors of the measured variables in both limbs at self-selected (SS) and fast (FS) walking speeds (N = 24). White bars represent data from SS and black bars represent data from FS. **(A)** AGRF normalized by body weight. **(B)** Trailing limb angle. **(C)** Ankle moment normalized by body weight. **(D)** Lever arm length for GRF. * *p* ≤ 0.05 and ** *p* ≤ 0.01.

The participants demonstrated a range of walking speeds (Table 1) and biomechanical measurements (Figure 4). Walking speed increased 26%. Significant increases were observed in all biomechanical variables (*p* < 0.01 for AGRF, *TLA*_*cop*_, *d,* and *p* < 0.05 for ankle moment) in both limbs.

**Table 1:** **Walking speeds and relative contributions to increases in propulsive force from changes in each variables**

**Subject**	**Age (yrs)**	**SS (m/s)**	**FS (m/s)**	**Relative contribution to paretic propulsion**				**Relative contribution to non-paretic propulsion**				**Handrail forces/BW**	
				**TLA**	***M***_***a***_	**mix**	***d***	**TLA**	***M***_***a***_	**mix**	***d***	**Vertical**	**Horizontal**
1	63	0.94	0.96	0.56	0	0	0.44	0.51	0.44	0.05	0	0.04	-0.01
2	65	0.70	0.78	0.23	0.66	0.11	0	0.39	0.58	0.03	0	0.06	-0.02
3	60	0.30	0.40	0.78	0.08	0.14	0	0.26	0.65	0.09	0	N/A	N/A
4	54	1.16	1.59	0.97	0	0	0.03	0.40	0.48	0.12	0	0	0
5	61	0.80	1.10	1	0	0	0	0.50	0.44	0.06	0	N/A	N/A
6	78	0.69	0.74	0.07	0.93	0	0	0	0	0	1	N/A	N/A
7	60	1.06	1.19	1	0	0	0	0.89	0.04	0	0.07	N/A	N/A
8	70	0.75	0.83	1	0	0	0	0.89	0	0	0.11	0.11	-0.01
9	70	0.47	0.69	0.83	0.12	0.05	0	0.93	0.05	0.02	0	0.07	0.07
10	55	0.45	0.78	0.72	0.11	0.17	0	0.71	0.18	0.11	0	0	0
11	63	0.27	0.47	0.65	0.08	0.27	0	1	0	0	0	0.09	-0.02
12	43	0.61	0.87	0.72	0.15	0.13	0	0.65	0.25	0.10	0	0.11	0.11
13*	58	0.61	0.65	0.57	0.37	0.06	0	0	0	0.21	0.79	0.19	0.18
14	71	1.16	1.36	0.51	0.46	0.03	0	1	0	0	0	0.04	0.04
15	55	0.74	1.01	0.64	0	0.36	0	0.79	0.18	0.03	0	0.10	-0.03
16	78	0.72	1.11	1	0	0	0	0.59	0.25	0.16	0	0.05	-0.02
17	61	0.80	1.14	1	0	0	0	0.90	0.06	0.04	0	0.05	0.05
18	62	1.13	1.37	1	0	0	0	1	0	0	0	0.12	0.12
19	55	0.88	1.10	0.71	0.21	0.08	0	0.34	0.63	0.03	0	0.03	0.03
20	62	1.01	1.11	0.83	0.11	0.06	0	0.66	0.20	0.05	0.09	0	0
21	71	0.88	1.23	0.18	0.77	0.05	0	0.73	0.21	0.06	0	0.12	0.12
22	56	1.13	1.46	0.97	0	0	0.03	1	0	0	0	0	0
23	47	0.42	0.60	0.82	0.01	0.17	0	0.57	0.27	0.16	0	0.10	-0.03
24*	25	1.51	1.68	1	0	0	0	1	0	0	0	0.05	-0.02
Mean	60	0.80	1.01	0.74	0.17	0.07	0.02	0.67	0.22	0.05	0.06	0.07	0.03
SD	11	0.30	0.34	0.28	0.28	0.10	0.09	0.28	0.22	0.05	0.21	0.05	0.06
max	78	1.51	1.68	1	0.93	0.36	0.44	1	0.65	0.16	1	0.19	0.18
min	25	0.27	0.40	0.07	0	0	0	0	0	0	0	0.00	-0.03

SS = self-selected walking speed, FS = fast walking speed, TLA = trailing limb angle, *M*_*a*_ = ankle moment, mix = interaction term, *d* = lever arm length. “N/A” denotes that handrail forces data not available.

Handrail forces were normalized by bodyweight. Positive values of handrail forces in the vertical and horizontal direction indicate forces pointing downwards and backwards, respectively.

*Subject#24 did not increase paretic propulsive force and subject#13 did not increase non-paretic propulsion during speed modulation. Thus, the relative contributions of the variables for these two subjects were not included in the overall averages in Table 1.

The relative contributions of the four components of Eq.5 to increases in propulsive force were quantified for each limb (Table 1). For the paretic limb propulsion, on average, the contributions of changes in TLA, ankle moment, lever arm length, and the interaction between TLA and ankle moment to increases in propulsive force were 74%, 17%, 2%, and 7%, respectively (Table 1). Thus, the ratio of the contribution of TLA versus ankle moment to increases in paretic propulsion was approximately 4:1. One subject (#24) did not increase paretic propulsive force during speed modulation. Eight subjects showed that the increases in TLA contributed more than 95% of increases in paretic propulsive forces and 11 subjects showed less than 2% contribution from the ankle moment to the increased propulsive force. Three subjects showed greater contribution from ankle moment (93%, 77%, and 66%) to propulsive force than from TLA.

For the non-paretic limb, on average, the contribution of changes in TLA, ankle moment, lever arm length, and the interaction between TLA and ankle moment to increases in propulsive force were 67%, 22%, 6%, and 5%, respectively (Table 1). Thus, the ratio of the contribution of TLA versus ankle moment to increases in non-paretic propulsion was approximately 3:1. One subject (#13) did not increase non-paretic propulsive force during speed modulation. Five subjects showed that increases in TLA contributed more than 95% of increases in non-paretic propulsive forces and 7 subjects showed less than 2% contribution from the ankle moment to the increased propulsive force. One subject (#6) had a minor increase in propulsive force with decreased lever arm length and no increase in TLA or ankle moment. Three subjects showed greater contributions from increases in ankle moment (58%, 65%, and 63%) than from increases in TLA.

## Discussion

In this study we found that the biomechanical-based model developed from able-bodied individuals (using ankle moment and TLA to predict propulsive force) over-estimated the propulsive force in stroke survivors. Thus, an enhanced model was developed and validated to describe the relationships between ankle moment, TLA (measured from the center of pressure), lever arm length between the GRF and the ankle joint, and propulsive force. The main finding was that individuals poststroke increase their propulsive force mostly by increasing TLA with relatively little contribution from ankle moment.

In contrast to our previous model developed from able-bodied individuals, the lever arm length of the GRF was included as a variable in the present model. Because the lever arm length can vary with the position of the ankle joint, the position of the COP, and the angle of the GRF vector (Figure 1), this length is likely to be different across individuals and to change with walking speeds. The present study showed that this length increased during speed modulation (Figure 4). In addition, rather than measure TLA from the great trochanter to the 5^th^ metatarsal, the present study measured to the foot’s COP. Measuring TLA to the COP allowed our model to capture the variance in COP position at late stance and therefore enhanced our model. For a population with a wide range in joint angles and COP positions, such as stroke survivors, variations across walking speeds and among individuals in these parameters can be large and, therefore, needed to be considered. Participants in this study showed a range of self-selected walking speeds from 0.27 to 1.51(m/s) and fast walking speeds from 0.4 to 1.68(m/s). Thus, the present model seemed to work for a wide range of walking speeds.

In agreement with previous studies, the present results showed that individuals poststroke increased TLA and peak AGRF at their fast walking speeds. Tyrell and colleagues observed increases in peak hip extension angle and peak TLA in the paretic limb when individuals poststroke progressively increased their walking speeds [8]. Similarly, increases in paretic peak TLA and AGRF during fast speed were also reported in a previous study of the effects of fast treadmill walking on poststroke gait [10]. Changes in TLA observed in this study were greater than the within-session minimal detectable change (1°) for this variable [11]. Also consistent with previous studies, increases in ankle plantarflexion moment were observed at the fast speed in the present study. Nadeau and colleagues found an increase in muscle utilization ratio, the ratio of the ankle plantarflexion moment used during gait to the maximal moment estimated from dynamometric measurements [12], at the fast walking speed in chronic stroke survivors [13]. However, in a study of the relationship between joint power and walking speeds in individuals poststroke, increases in paretic ankle plantarflexion power at fast walking speed were only significant for the higher functioning group [14]. Interestingly, although our results showed an average increase in ankle moment, 10 of 24 subjects did not increase their paretic ankle moment and 7 subjects did not increase their non-paretic ankle moment at the fast speed compared with their self-selected speed.

In the paretic limb, the majority of the change in propulsive force was contributed from the change in TLA (74%); relatively little contribution from ankle moment (17%) was observed in the paretic leg during speed modulation (Table 1). This finding is similar to our previously reported results in able-bodied individuals that showed that TLA was the major contributor (66%) to increases in propulsive force during speed modulation [9]. One possible explanation of this greater contribution from TLA could be due to the weakness or inability to modulate the force in the paretic ankle plantarflexor muscles in stroke survivors. Jonkers and colleagues found that lower functioning hemiparetic subjects engaged excessive plantarflexor power generation at SS walking speeds and therefore no further increase was revealed during the fast walking speed condition [14]. The inability to modulate ankle plantarflexor muscles in individuals poststroke may only allow them to modulate TLA to increase propulsive force. Our results showed a wide variation of contributions from the paretic ankle moment to increases in propulsive force across individuals. Interestingly, in contrast to what we had anticipated, the average walking speed for the 10 subjects who showed no contribution from ankle moment to the increase in propulsion was substantially higher than the average walking speed of the rest of 14 subjects (1.0 versus 0.66 m/s). In fact, subjects 18 and 22 increased both paretic and non-paretic propulsion without increasing ankle moment, yet both of these individuals were amongst the 5^th^ fastest walkers. Thus, individuals who adopted the TLA strategy to increase propulsive force were not only limited to slower ambulators.

In the non-paretic limb, the contribution from TLA (67%) to the increase in propulsive force was also greater than the contribution from ankle moment (22%). Thus, on average, the ratio of the contribution of TLA versus ankle moment to the increase in propulsive force was about 3:1 in the non-paretic limb and 4:1 in the paretic limb. This ratio in the non-paretic limb is closer to the ratio reported in able-bodied individuals (2:1). In addition, compared to the paretic limb, fewer subjects adopted the strategy that uses TLA alone to increase propulsive force on their non-paretic limbs. For individuals post-stroke, the rate of force development and voluntary activation of the plantarflexor muscle has been shown to be considerably reduced in the paretic limb compared to the non-paretic limb [15]. Investigations on whether improving paretic ankle plantarflexor strength will modify the strategy adopted to increase propulsion would provide insight into the reason why individuals select particular strategies to increase propulsion.

Although changing TLA alone may allow for increasing propulsive force without requiring additional force to be generated from the ankle plantarflexor muscles, the lack of push-off force may eventually lead to more mechanical work being needed to complete the redirection of the COM velocity during the step to step transition [16,17]. Using a simple walking model, Kuo studied the mechanical energy needed to overcome energetic losses incurred at heel strike and found that an impulse applied to the stance foot immediately before heel strike is four times less costly than driving the stance leg via torque at the hip [18]. Thus, the excessive reliance on increasing TLA and the concomitant increase in hip torque alone, rather than also increasing ankle moment to increase propulsive force, may demand more mechanical work for individuals poststroke. Future investigation to determine if greater mechanical work is actually observed for individuals who depend on TLA alone to increase propulsive force during gait is needed. It is worth noting that although the average increases in TLA reported from this study were comparable to the previously reported values in able-bodied individuals, the TLA at self-selected and fast walking speeds in this study were still relatively small compared with able-bodied individuals [9]. That is, stroke survivors still need to improve TLA to restore normal walking pattern. However, for individuals who do not increase their ankle moment to increase propulsion, gait interventions targeting improving TLA may lead to a more energy inefficient gait. Thus, the capacity to increase ankle plantarflexion moments may be a criteria to evaluate individuals who will benefit most from interventions that increases walking speed or propulsion.

One factor that the present study did not measure is risk of falling. Although physiological constraints such as muscle strength or energy cost are important factors in gait, preference of strategy may be influenced by fear of falling [19]. For example, if increasing ankle moment to increase walking speed could lead to increase in risk of falling, individuals poststroke may avoid this strategy regardless of metabolic efficiency. Thus, future investigation measuring balance in conjunction with TLA and ankle moment is important for understanding the mechanism individual select to increase propulsion and for directing gait intervention.

There were limitations in this study. First, our model was not applicable for individuals who did not position their feet posterior to their body at terminal stance. A foot position anterior to the COM would result in a negative TLA. Based on our model, a negative TLA would produce a posterior ground reaction force and therefore generate a braking force rather than a propulsive force. Thus, individuals with negative TLA were excluded from this study. Second, subjects participating in this study were allowed to hold onto the handrails. The use of handrails could influence gait patterns and force distribution. For example, subjects could use the handrail to support part of their body weight and therefore decrease the force needed from their legs. In addition, the use of a handrail may cause individuals to lean their body toward the handrail rather than staying upright. This body leaning may affect the angle of the ground reaction force without being captured by our model as TLA and ankle moment did not account for upper body movements. Thus, the accuracy of our model could be affected by handrail use. However, verbal instructions on using the handrail as minimal as possible were provided during data collection. Our results showed that the handrail forces were subject-specific and were not correlated to mechanisms of increasing propulsion (see Table 1). For example, subjects#12, #18 and #21 used similar handrail forces but very different lower extremity strategies to increase propulsion. Another potential limitation in this study was the sensitivity of our model. Three participants had small increases in walking speed from self-selected to fast (subjects #1, #6, and #13). Each of these subjects showed large contributions from changes in the lever arm length. Thus, our model may not be suitable for analyzing very small increases in walking speeds. Finally, the present study did not have an age-matched control group and therefore could not exclude the effect of age on mechanisms to increase propulsion. Thus, future studies comparing individuals poststroke and age-matched able-bodied individuals are needed. However, based on the data presented in Table 1, there was no obvious relationship between subjects’ ages (range: 25-78 years) and the mechanism for increasing propulsion.

## Conclusions

This is the first study that quantified the relative contribution of ankle moment and TLA to the increase in propulsive force during poststroke gait. By enhancing a previously developed biomechanical-based model, the present results showed that individuals poststroke increase propulsive force mainly by changing TLA for both the paretic and non-paretic limbs. In addition, the present model has the potential application to determine the mechanism used to improve propulsive force pre and post intervention.

## Abbreviations

AGRF: Anterior ground reaction force
COP: Center of pressure
COM: Center of mass
GRF: Ground reaction force
TLA: Trailing limb angle
SS: Self-selected
FS: Fast
